# Different Trajectories, Stable Links: Parental Worry and Child Internalizing Symptoms Over Time

**DOI:** 10.1007/s10964-025-02237-1

**Published:** 2025-08-22

**Authors:** Shannon Taflinger, Marcus Eisentraut

**Affiliations:** University of Cologne, Department of Sociology and Social Psychology, Cologne, Germany

**Keywords:** Parental worry, Child internalizing symptoms, Longitudinal trajectories, Bidirectional effects, Parental anxiety, Child mental health

## Abstract

While research has investigated the relation between (general) parent anxiety and child mental health outcomes, parental worry specific to one’s children has often been overlooked. Therefore, this study examines longitudinal dynamics between parental worry and child internalizing symptoms. Data are from waves 3–13 (2010–2020) of the German Family Panel (pairfam). Parental worry and child symptoms were reported by parents and children, respectively, every two years. The sample includes children ages 8–15 (N_boys_ = 667, N_girls_ = 593) and their parents (N_fathers_ = 290, N_mothers_ = 646) living in Germany. Results show that initial levels of parental worry and child internalizing symptoms (ages 8–9) are related and remain stable over time, however, parent and child trajectories are not related. While children’s symptoms tend to decrease, parental worry also decreases with little variation. The results do not provide evidence for bidirectional influences on each other’s trajectories.

## Introduction

Extensive evidence demonstrates the broader impact of (general) parental anxiety on child mental health outcomes, such as anxiety and depression (Lawrence et al., 2019). Nevertheless, less attention has been given to parental worries specific to one’s children– such as fears that something bad could happen to them – which is theorized to play a mediating role in explaining the relationship between parental and child anxiety (Fisak et al., 2012). Moreover, most research to date has been cross-sectional; hence, it is unclear how these relations evolve across development. Taking a longitudinal perspective is especially important given the possibility of co-occurring trajectories (Griffith et al., 2023) as well as the possibility that children also shape their parents (Pardini, 2008). This study examines the longitudinal relations between parental worry and child internalizing symptoms, specifically testing whether the initial levels and trajectories of parental worry and those of child internalizing symptoms are related and whether the relation between parental worry and child internalizing symptoms changes as the child ages. To answer these questions, this study utilizes the German Family Panel (pairfam) release 14.0 (Brüderl et al., 2024), focusing on the relations between parents and their children from late childhood through adolescence (ages 8–15).

### Parental Worry and Child Internalizing Symptoms

Worry is characterized by a stream of negative thoughts and images as part of repeated attempts to solve an issue with an uncertain outcome. This cognition is often described as “uncontrollable”, given the difficulties individuals experience in stopping or preventing worrying from occurring (Borkovec et al., 1983; Mathews, 1990). Among parents, common worries include academic achievement and the well-being of their children (Fisak et al., 2012). As individuals high in anxiety are more likely to perceive aspects of their environment as threatening (Lester et al., 2009), worry about one’s children may lead parents to excessively regulate their children’s activities to lower their own distress (L. B. Jones et al., 2021; Turner et al., 2003). Such overcontrolling parenting, in turn, likely fosters an external locus of control, namely the belief that events are not within their control, as well as feelings of helplessness and anxiety (Chorpita & Barlow, 1998).

The relationship between parental anxiety, parental control, and child mental health outcomes has been widely studied with several meta-analyses synthesizing existing evidence. Firstly, a meta-analysis demonstrated that parental anxiety is associated with increased behavioral control measured via observation. However, this only occurred in parents of older children and adolescents, but not in younger children (Van Der Bruggen et al., 2008). Similarly, another meta-analysis showed that maternal anxiety is related to overprotective parenting (L. B. Jones et al., 2021) and that controlling parenting has small effects on child anxiety and depression (McLeod, Weisz, et al., 2007; McLeod, Wood, et al., 2007). Meanwhile, autonomy granting, supporting the child making their own decisions and self-expression, is negatively related to internalizing symptoms, which include anxiety, depression, and psychosomatic symptoms (i.e., stomachaches) (Pinquart, 2017).

Though parental worry about one’s children has received less empirical attention than parental anxiety and overprotective parenting, existing evidence suggests it may play a meaningful role in shaping child mental health outcomes. A study using a non-representative sample of caregivers from the US and UK showed that parental worry mediates the effect of parental (general) anxiety on child anxiety (Fisak et al., 2012). While the analyses controlled for demographic covariates, e.g., ethnicity, caregiver age, child age, the effects of these contextual factors were not reported in the results. Moreover, recent research from China examined how general parental worry influences child anxiety via anxious childrearing among young children (Zhou & Li, 2022). Anxious rearing behavior was operationalized using a scale that includes both worry about one’s child, e.g., worry about the child making a mistake, and parenting behavior, e.g., warning the child of possible dangers, restricting the child’s activities. The study found that maternal general worry and paternal general worry affected child anxiety one year later via maternal anxious rearing behavior; however, paternal anxious childrearing did not play a mediating role. Several demographic variables were tested and not found to be related to the outcome variables, e.g., paternal education, family monthly income, child age. The exception was maternal education, which was positively related to maternal anxious rearing behaviors and included as a control variable.

Due to the high comorbidity between anxiety and other mental health diagnoses (Gorman, 1996), other research has examined depression as an additional outcome variable. Cross-sectional evidence from a small Romanian sample of mother-child dyads shows that (general) maternal worry influences both child depression, generalized anxiety, and social anxiety via child worry, though the study does not specify whether any covariates were controlled for (Pasarelu et al., 2015). Given the limited research on how parental worry specifically about one’s child influences child outcomes, further empirical investigation is necessary to better understand its implications for child mental health.

### Linked Trajectories

While theories on parental anxiety and child outcomes tend to center around the causal effects of the parents on the child (Chorpita & Barlow, 1998; Wood et al., 2003), the potential for children to shape their parents has recently received increasing attention (Pardini, 2008). The possibility of reciprocal influences was originally brought to attention in the late 1960s (Bell, 1968), and later theories have similarly taken a transactional perspective, which explicitly recognizes that the child plays a role in shaping their environment (Sameroff, 1975). Similarly, the bioecological model argues in favor of bidirectional influences, going a step further to emphasize temporal aspects in the development of children, which should be accounted for in empirical tests of parent-child influences (Bronfenbrenner & Morris, 2007). This perspective aligns with the life course principle of “linked lives,” which emphasizes how the lives of family members are interconnected, with each member’s experiences influencing others over time (Bengtson et al., 2011).

Bidirectionality is similarly plausible in the relation between parental worry and child mental health outcomes, as child emotional distress could in turn prompt increases in worry and thereby excessive parental involvement in the hopes of alleviating the anxiety or helping the child accomplish a task (Bögels & Brechman-Toussaint, 2006). Some research has begun to examine reciprocal effects using cross-lagged panel models and found support for reciprocal effects between parent and child mental health in terms of both psychological distress (Ge et al., 1995) and internalizing symptoms (Villarreal & Nelson, 2018). A more recent study using a random intercept cross-lagged panel model, which accounted for both within- and between-family variation and included multiple perspectives from both children and parents, found that maternal overprotection, as reported by children, led to an increase in child anxiety a year later, as reported by parents. However, the study found no evidence of reciprocal effects when parents reported their own overprotection and children reported their own anxiety, respectively (Jian et al., 2025).

Another area that has been receiving increased attention is the potential for parents and children to not only mutually affect each other at specific points in time, but also shape each other’s trajectory of change (cf. Griffith et al., 2023). For example, a study of children aged 8–16 in the USA used parallel latent growth curve modeling to demonstrate that there is a positive relation between parent and child stress trajectories and that the effect of parental depressive symptoms on child depressive symptoms is mediated by child stress (Griffith et al., 2023). In this study, stress was operationalized as exposure to stressful life events, and these associations did not differ by gender.

Moreover, a longitudinal study of Australian mothers and children found that maternal mental health when the child is 4–5 years predicted an increasing trajectory of child internalizing symptoms through middle childhood. Although internalizing problems rose on average for all children, both the initial levels and the rate of increase were higher for girls; meanwhile, socioeconomic status did not play a role (Wang et al., 2018). Similarly, a study from the USA showed that higher maternal anxiety was related to higher initial child anxiety at age 14 and increases in child internalizing symptoms through 18 years old, while friendship quality had a buffering effect on the relation. They found no difference in the slope of internalizing symptoms between girls and boys (Havewala et al., 2019).

Finally, an exploratory study from the UK applied latent class growth models to model the joint development of maternal and paternal psychological distress and child internalizing symptoms trajectories from ages 3 to 14. Four distinct trajectory classes were identified, distinguished primarily by mean levels rather than rates of change. Across all classes, maternal psychological distress, paternal psychological distress, and child internalizing symptoms tended to increase over time. The symptoms tended to increase across all groups (Zhu et al., 2023), highlighting the need for further analyses to understand whether such increasing trajectories are statistically independent or co-develop over time.

### Changes throughout Development

There are competing theoretical propositions about how the relation between parenting and child anxiety changes over the course of development (Wei & Kendall, 2014). Patterson (1995) suggests parent-child dynamics can be characterized by cumulative effects and escalation as family interactions are contingent on the behavior of others and one’s own behavior is shaped by the behavior of the other person in the social interaction. While this research originally examined the development of antisocial behavior, such expectations can be applied to parental worry and child internalizing symptoms. For example, if a parent responds to the child’s anxious-avoidance behavior with increased accommodation, e.g., allowing the child to skip a test, the child may learn that demonstrating such anxious behaviors is a strategy to avoid situations perceived to be threatening, and engaging in them more frequently and with increased intensity in the future. Such accommodation has been shown to mediate the relation between maternal and child anxiety (J. D. Jones et al., 2015). It is also similar to the “FEAR effect”, namely the family enhancement of avoidant and aggressive responses, whereby parents of anxious children reward and reinforce avoidant behavioral strategies when confronted with a potential threat (Dadds et al., 1996). From a socialization perspective, others have argued that parental effects on the child decrease as the child ages (Chorpita, 2001) because children spend more time with peers and less time with families during adolescence (Larson & Richards, 1991).

Empirical research examining the effect of parental anxiety and parenting behaviors on child mental health outcomes is often tested via meta-analyses and yields mixed results. Two meta-analyses found no moderation of the relation between parenting and child anxiety by mean child age (McLeod, Wood, et al., 2007) as well as between parenting and child anxiety, depression, and internalizing symptoms (Yap & Jorm, 2015). Meanwhile, a meta-analysis focusing on paternal control measured via observation found that the relation between parental control and child anxiety and between parental anxiety and parental control increases with the mean child age in the study (Van Der Bruggen et al., 2008).

Additionally, a study using individual-level data from a sample of Dutch children (ages 8–12) and adolescents (ages 13–18) found that the associations between parental overcontrol, parental autonomy granting, and child anxiety tend to be stronger in childhood compared to adolescence (Verhoeven et al., 2012). However, the direction and strength of moderation varied depending on age group and parental behavior examined. Maternal and paternal overcontrol were positively related to anxiety in children. Meanwhile, autonomy granting was positively associated with anxiety in children under 10 but was unrelated to anxiety in those over 10. Among adolescents, paternal overcontrol was positively associated with child anxiety, and the effects were strongest when they were older than 15. Neither maternal overcontrol nor parental autonomy granting was related to anxiety in adolescents. Additional moderation analyses showed that the effects of parental overcontrol and autonomy granting on child anxiety were similar for boys and girls. In sum, there are competing theoretical perspectives that can be used to explain how the relation between parental worry and child internalizing symptoms changes over time, and existing empirical evidence is mixed.

## The Present Study

To date, parental worry about one’s children has been overlooked in the examination of child mental health outcomes, and studies that exist tend to be cross-sectional. The current study addresses the paucity of longitudinal research on parental worry by examining whether parental worry and child internalizing symptoms co-develop from early childhood through adolescence. As worry about one’s children may lead to overprotective parenting behaviors, which negatively affect the child’s mental health, the initial levels of parental worry and child internalizing symptoms are expected to be positively related (Hypothesis 1). Given the likely bidirectional relationship—where parental worry contributes to parenting behaviors that increase child internalizing symptoms, and elevated child symptoms, in turn, heighten parental worry—it is expected that the trajectories of parental worry and child internalizing symptoms are related (Hypothesis 2) and that initial levels of parental worry (child internalizing symptoms) are associated with increases rate of change in child internalizing symptoms (parental worry) over time (Hypothesis 3_a-b_). Furthermore, existing theoretical perspectives offer contrasting predictions about whether the association between parental worry and child internalizing symptoms strengthens or weakens over time: one framework suggests mutual, escalating influences, while the other emphasizes a declining role of parents relative to peers during adolescence. Accordingly, it is expected that the covariance between parental worry and child internalizing symptoms increases or decreases as the child ages (Hypothesis 4).

## Method

### Data and Sample

This study uses data from the German Family Panel (pairfam), release 14.0 (Brüderl et al., 2024). Pairfam is a multi-actor cohort panel, which includes anchor (parent) and child questionnaires (ages 8–15). Data collection occurred yearly from 2008/2009 to 2021/2022, beginning with a random sample of approximately 12,000 anchors. The child survey first began in wave 2 of the main survey (2009/2010). The target population was approximately German-speaking individuals living in private households in Germany belonging to one of four age cohorts: born in 1971–73 (aged 35–37 in wave 1); 1981–83 (aged 25–27 in wave one); 1991–93 (aged 15–17 in wave one); and 2001–2003 (aged 5–7 in wave 1; added in the refreshment sample in wave 11 at ages 15–17). Sampling occurred in a two-step process. First, municipalities were selected, with an oversampling of municipalities from East Germany. Then, individuals from the chosen municipalities were selected based on local population registers. Anchors (parents) needed to provide consent for their children to participate in the interviews. Coverage rates, the total percentage of eligible children that participated in the survey, ranged from 55–73% over the waves. Interviews were conducted via Computer-Assisted Personal Interviewing (CAPI) and shifted to Computer-Assisted Telephone Interviewing (CATI) during the COVID-19 pandemic (Brüderl, Schmiedeberg, et al., 2023).

To be included in this study’s analytical sample, parents and children had to provide non-missing answers on at least one of the items of the variables parental worry and child internalizing symptoms at a minimum of three timepoints. Given that the parental worry variable is only assessed every other wave starting in wave 3, children were combined into four age groups (ages 8–9; 10–11; 12–13; 14–15). The sample was restricted to pairfam’s target child sample, namely children ages 8–15. One observation was deleted as the child’s gender changed in one wave. Although the data were collected yearly, some children were the same age in two waves due to slight differences in the timing of the data collection. Therefore, age was recoded as years since the age at the first observed wave. Observations whose adjusted age fell outside this age range were also not included in the final sample (22 observations). Because some respondents joined the panel after the child was older than ages 8–9 or dropped out before the child was 14–15, the highest rates of both parent and child participation were when the child was in the age groups 10–11 and 12–13.

The final sample consists of 1260 parent-child dyads (936 unique parents due to siblings in the dataset) and 4560 observations (see Table 1 for sample descriptives). The sample is roughly split equally between girls (47.1%) and boys, while mothers were overrepresented (69.4%). Approximately half of the parents had a university degree. Most parents were born in Germany (89.0%), including the former eastern German Democratic Republic. Approximately 18% of parents were from the East Germany oversample. Most parents had only one child in the sample (68.9%), a smaller portion had two children (28.0%), and very few had three (2.7%) or four (0.4%).

**Table 1:** Descriptive information on sample parent-child dyads (*N*_*parents*_ = 936; *N*_*children*_ = 1260)

	Mean/proportion (Standard deviation)	Minimum	Maximum	Missing values (proportion)
*Sample description: parents*				
Gender				0
Woman	0.690			
Man	0.310			
Age (in years)	40.424 (4.842)	27	50	0.103
Highest level of education	5.228 (1.662)	1	8	0
Monthly net household income	3529.623 (1553.214)	0	12,000	0.153
Total number of children	2.294 (0.988)	1	8	0.103
Number of children in sample	1.346 (0.553)	1	4	0
Relationship status				0.113
No cohabitating partner	0.055			
Cohabitating partner	0.945			
Currently living in East Germany				0.103
No	0.632			
Yes	0.368			
East German oversample (DemoDiff)				0
No	0.819			
Yes	0.181			
Migration status				0
German-born	0.890			
Immigrant	0.110			
*Sample description: children*				
Child gender				
Girl	0.471			0
Boy	0.529			
Age (in years)	11.409 (2.130)	8	15	0.077
Child age wave 1 (pairfam)	4.460 (2.519)	0	9	0

The sample consists of 1260 parent-child dyads representing 936 unique parents, as some parents have multiple children in the dataset. Monthly net household income values over 12,000 were determined to be potential data quality issues and recoded as missing. Child age is the reported age. The high percentage of missing values for time-varying variables is generally due to participation rates, rather than item non-response.

Attrition analyses were conducted to assess the extent to which inclusion in the final sample was influenced by parental worry, child internalizing symptoms, and potential covariates. The analyses compared parent-child dyads in the final sample to those in which the child answered the child survey at least once and those whose parents were not from the refreshment sample, as refreshment sample cases a priori could not reach the threshold of a minimum of three observations. Analyses were tested using a logit model with cluster robust standard errors (see Table S1). The attrition analyses show that controlling for all other variables, child age during first participation (*OR* = 0.380, *p* < 0.001); being a mother (reference group is fathers, *OR* = 1.443, *p* < 0.001); child age during first pairfam wave (*OR* = 1.316, *p* < 0.001); and parental education (*OR* = 1.103, p = 0.001) were related to inclusion in the final analyses. There was a slight tendency for parents with lower levels of parental worry (*OR* = 0.909, p = 0.061) and boys (*OR* = 0.873, *p* = 0.096) to be less likely to participate; however, differences were not statistically significant at the level of 0.05. There were no meaningful differences in parent migration status, inclusion in the East German oversample, first recorded child internalizing symptoms, parental monthly net household income, parent relationship status, the number of children in the household, and living in East Germany.

### Measures

#### Parental Worry Specific to the Child

Parental worry specific to the child was assessed with a 3-item scale, measured from the parent perspective (cf. Engfer, 1984) and referred to as “Overprotection” in the pairfam Scales Manual (Brüderl et al., 2024). Parents were asked, “If you think of your child(ren), to what extent do the following statements apply to you”. The items were (1) “I am always worrying that something could happen to my child(ren)”; (2) “I am always asking myself if I am doing the right thing for my child(ren).” (3) “Sometimes I cannot sleep because I imagine something could happen to my child(ren)”. Response items ranged from 0 (“Not at all”) to 4 (“Absolutely”). This scale was assessed in odd waves beginning in wave 3. Cronbach’s α was 0.762, indicating acceptable reliability.

#### Child Internalizing Symptoms

Child internalizing symptoms is a scale of five items from the Strengths and Difficulties Questionnaire (cf. Goodman, 1997) assessed from the child’s perspective. Children were asked to what extent a series of statements were true over the past six months. The items were (1) “I get a lot of headaches, stomachaches or sickness”; (2) “I worry a lot”; (3) “I am often unhappy, depressed or tearful”; (4) “I am nervous in new situations. I easily lose confidence”; (5) “I have many fears. I am easily scared”. The response options were 0 (“Not true”), 1 (“Somewhat true”), and 2 (“Certainly true”). This scale was assessed in waves 2–14. Cronbach’s α was 0.654, indicating acceptable reliability.

### Analytic Strategy

#### Parallel Latent Growth Model

**Fig. 1:**
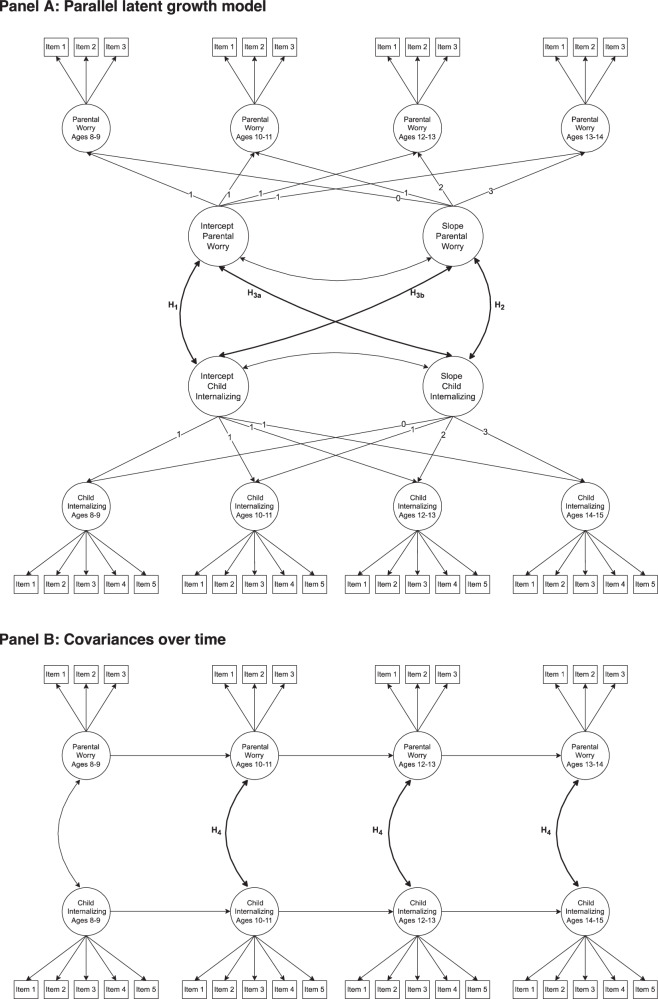
**A** depicts a parallel latent growth model that tests the relations between the latent intercepts and slopes (Hypotheses 1–3, in bold). **B** shows a model that tests the stability of the covariances between parental worry and child internalizing symptoms over time (Hypothesis 4, in bold)

Hypotheses 1–3 were tested using a parallel latent growth model (PLGM) using Mplus, Version 8.11 (see Fig. 1 for a conceptual model). Although many studies have examined bidirectional effects between parents and children using cross-lagged panel models (e.g., Allmann et al., 2022; Ge et al., 1995; Van Zalk et al., 2018), there are many recent criticisms of this method. Cross-lagged panel models have been shown to yield biased effects because they do not take into account stable between-person differences (Lucas, 2023). Extensions of the model, such as the random effects cross-lagged panel model (Hamaker et al., 2015), have similarly been shown to be biased when the process under examination is not in equilibrium (Andersen, 2022). Conversely, the PLGM models the associations between initial levels and rates of growth using structural equation modeling (SEM) (Preacher et al., 2008). Thereby, it is subject to fewer assumptions but can still be used to test the potential linked lives aspect of parental worry and child internalizing symptoms by modeling the associations between initial levels and rates of growth (cf. Sticca & Perren, 2015).

First, latent growth models (LGMs) were tested for parental worry and child internalizing symptoms. Each model had two components: measurement and structural. In the measurement part, the items of each construct were loaded onto wave-specific latent variables. In the structural part, each of the wave-specific latent variables was loaded onto a latent intercept and slope. The latent intercept represents the parent worry and child internalizing symptoms at ages 8–9, while the latent slopes represent the average change in parental worry and child internalizing symptoms over a two-year period, such that one unit reflects the average change over two years. Measurement invariance was then tested, and both parental worry and child internalizing symptoms demonstrated metric and scalar invariance. This satisfies the necessary conditions for meaningfully interpreting latent means and, therefore, also growth trajectories (Putnick & Bornstein, 2016).

Second, a PLGM model was tested that includes covariances between the latent intercepts and slopes. The model tested whether the initial levels of parental worry and child internalizing symptoms covary, whether their slopes covary, and whether the level of parental worry (child internalizing symptoms) at ages 8–9 predicts the rate of change in child internalizing symptoms (parental worry). An additional model included covariates.

#### Covariances Over Time

To test Hypothesis 4, an analysis was conducted to examine whether the covariances between parental worry and child internalizing symptoms change over time. The procedure began with the specification of a measurement model in which parental worry and child internalizing symptoms were loaded onto wave-specific latent variables. In the next step, covariances between parental worry and child internalizing symptoms were added, along with autoregressive paths for both parental worry and child internalizing symptoms from the preceding wave. To assess whether the strength of the associations changed over time, the covariances were constrained across three age groups (10–11, 12–13, and 14–15 years) to be equal, and model fit indices were compared to the unconstrained model. A significant reduction in model fit would suggest that the covariances vary across developmental periods.

#### Model Specification and Control Variables

All models used cluster-adjusted standard errors to account for the clustering of children within parents, i.e., siblings. To handle missing data, the models applied full information maximum likelihood (FIML). Due to the skewedness of the variable child internalizing symptoms, the models used maximum likelihood with robust standard errors (MLR, Maydeu-Olivares, 2017).

All models were tested controlling for likely confounders, variables that could potentially affect both parental worry and child internalizing symptoms. For example, previous research has shown that trajectories differ by child gender (Katsantonis, 2024; Leve et al., 2005), and socioeconomic status plays a role in shaping child anxiety (Letourneau et al., 2013); hence, these variables were included as covariates. The specific control variables tested were the child’s age in wave one of pairfam (potential cohort effects), the child’s gender, the parent’s gender, the parent’s highest level of education achieved (ISCED-97) as a continuous variable, logged net household income, the number of children in the parent’s household, whether or not the parent currently lives in East Germany, the relationship status of the parent (indicates whether the parent is currently living with a partner), sample (indicates whether the parent was recruited in the East Germany oversample), and the migration status of the parent (immigrant or German-born). Household income from 12 observations with reported monthly income over 12,000 euros were identified as outliers and, therefore, recoded as missing.

In order to preserve model stability, only control variables that showed significant predictive power for parental worry and child internalizing symptoms in preliminary models were included in the final parallel latent growth model. These include the parent’s education level, parent’s gender, child’s gender, and child’s age at wave 1 of pairfam. In the second model, which investigated changes in covariances between latent factors over time, no control variables were included. This decision was based on preliminary analyses and reflects concerns that the inclusion of covariates could introduce partial correlations, thereby obscuring the true developmental associations between parental worry and child internalizing symptoms.

## Results

### Descriptives

As depicted in Table 2, the most frequently endorsed parental worry item was worrying about something bad happening to their children, while the least frequently endorsed was difficulty sleeping due to worrying about one’s children. Parental worry items were slightly right-skewed, indicating parents more often reported low values than would be expected with a normal distribution. The mean values of the parental worry items appear to slightly decrease over time.

**Table 2:** Descriptive statistics for study variables (*N* = 1260)

	Time (age groups)											
	8–9 years old (baseline)			10–11 years old			12–13 years old			14–15 years old		
	Mean	SD	Missing	Mean	SD	Missing	Mean	SD	Missing	Mean	SD	Missing
*Parental worry*												
(1) Worry	1.812	1.192	0.168	1.788	1.179	0.007	1.703	1.168	0.004	1.649	1.164	0.239
(2) Questioning	1.758	1.104	0.168	1.695	1.081	0.007	1.591	1.069	0.006	1.504	1.089	0.239
(3) Cannot sleep	0.731	1.008	0.169	0.708	0.993	0.008	0.715	0.995	0.005	0.745	1.033	0.239
*Child internalizing symptoms*												
(1) Psychosomatic	0.459	0.619	0.236	0.426	0.606	0.025	0.376	0.592	0.014	0.346	0.588	0.308
(2) Worry	0.749	0.671	0.241	0.725	0.650	0.026	0.676	0.645	0.014	0.767	0.657	0.308
(3) Unhappy	0.427	0.573	0.241	0.329	0.518	0.028	0.254	0.474	0.014	0.273	0.519	0.308
(4) Nervous	0.574	0.648	0.253	0.530	0.623	0.029	0.530	0.621	0.018	0.602	0.647	0.308
(5) Fear	0.540	0.636	0.237	0.467	0.586	0.025	0.362	0.551	0.014	0.349	0.555	0.310

The range of the parental worry scale is 0–4, while the range of the child internalizing symptoms scale is 0–2. Standard deviation (SD). Missing values are shown in proportions. The higher proportion of missing values in the 8–9 group and 14–15 group is largely due to beginning the survey at later waves (in the ages 8–9 group) and participant drop out or not reaching ages 14–15 before the end of the data collection period, rather than item non-response.

Among children, the most frequent internalizing symptom reported by children was worrying, while the least frequently reported symptom was unhappiness or sadness. Child internalizing symptoms were quite positively skewed, and similar to parental worry, the mean items of child internalizing symptoms decreased slightly over time. Pairwise correlations of the items of the parental worry and child internalizing symptoms scales tend to be positively related (see Table 3 for wave 1 correlations).

**Table 3:** Bivariate correlations of parental worry and child internalizing symptoms (Wave 1)

	1)	2)	3)	4)	5)	6)	7)	8)
*Parental worry*								
(1) Worry	1.000							
(2) Questioning	0.494^***^	1.000						
(3) Sleep	0.572^***^	0.446^***^	1.000					
*Child internalizing symptoms*								
(4) Nervous	0.051	0.104^**^	0.059	1.000				
(5) Headaches	0.089^**^	0.094^**^	0.042	0.148^***^	1.000			
(6) Fear	0.089^**^	0.089^**^	0.064^*^	0.259^***^	0.177^***^	1.000		
(7) Unhappy	0.086^**^	0.116^***^	0.022	0.193^***^	0.299^***^	0.417^***^	1.000	
(8) Worry	0.153^***^	0.149^***^	0.113^***^	0.212^***^	0.235^***^	0.314^***^	0.338^***^	1.000

^*^p < 0.05, ^**^p < 0.01, ^***^p < 0.001

### Latent Growth Models

The latent growth models for both parental worry and child internalizing symptoms fit the data well (see Tables S2–3 and Figs. S1–2). The analysis began with the construction of the measurement model. For both parental worry and child internalizing symptoms, the first item was fixed. The loadings of the parental worry latent variable for items two and three were moderate and statistically significant (λ_2_ = 0.759, λ_3_ = 0.685, *p* < 0.001). Meanwhile, the loadings for child internalizing symptoms were quite strong and statistically significant (λ_2_ = 0.934, λ_3_ = 1.249, λ_4_ = 1.130, λ_5_ = 1.392, *p* < 0.001). Next, the structural components of the model were added, namely the latent intercepts and latent slopes. The variance of the latent intercept in the parental worry model was statistically significant (φ = 0695, *p* < 0.001) and the mean of the latent slope was negative and statistically significant (μ = −0.063, *p* < 0.001), however, the variance of the slope was statistically insignificant (φ = 0.008, *p* = 0.351). Hence, initial levels of parental worry were characterized by substantial between-person variation, while the trajectories were marked by a slight decreasing trend and lack of between-person variation. The covariance between the latent intercept and slope was statistically insignificant (*B* = −0.004, *p* = 0.812); thus, the level of parental worry at child ages 8–9 was not related to differences in parental worry trajectories.

The variance of the latent intercept in the child internalizing symptoms model was statistically significant (φ = 0.048, *p* < 0.001). The mean of the latent slope was slightly negative (μ = −0.017, *p* = 0.004), and the variance of the slope was statistically significant (φ = 0.007, *p* < 0.001). These findings indicate that there was inter-person variation in the initial states and trajectories of child internalizing symptoms, and children tended to report fewer internalizing symptoms over time. The covariance between the latent intercept and slope was negative and statistically significant (*B* = −0.007, *p* = 0.006); hence, children with high internalizing symptoms at ages 8–9 had more substantial decreases over time.

### Parallel Latent Growth Models

**Fig. 2:**
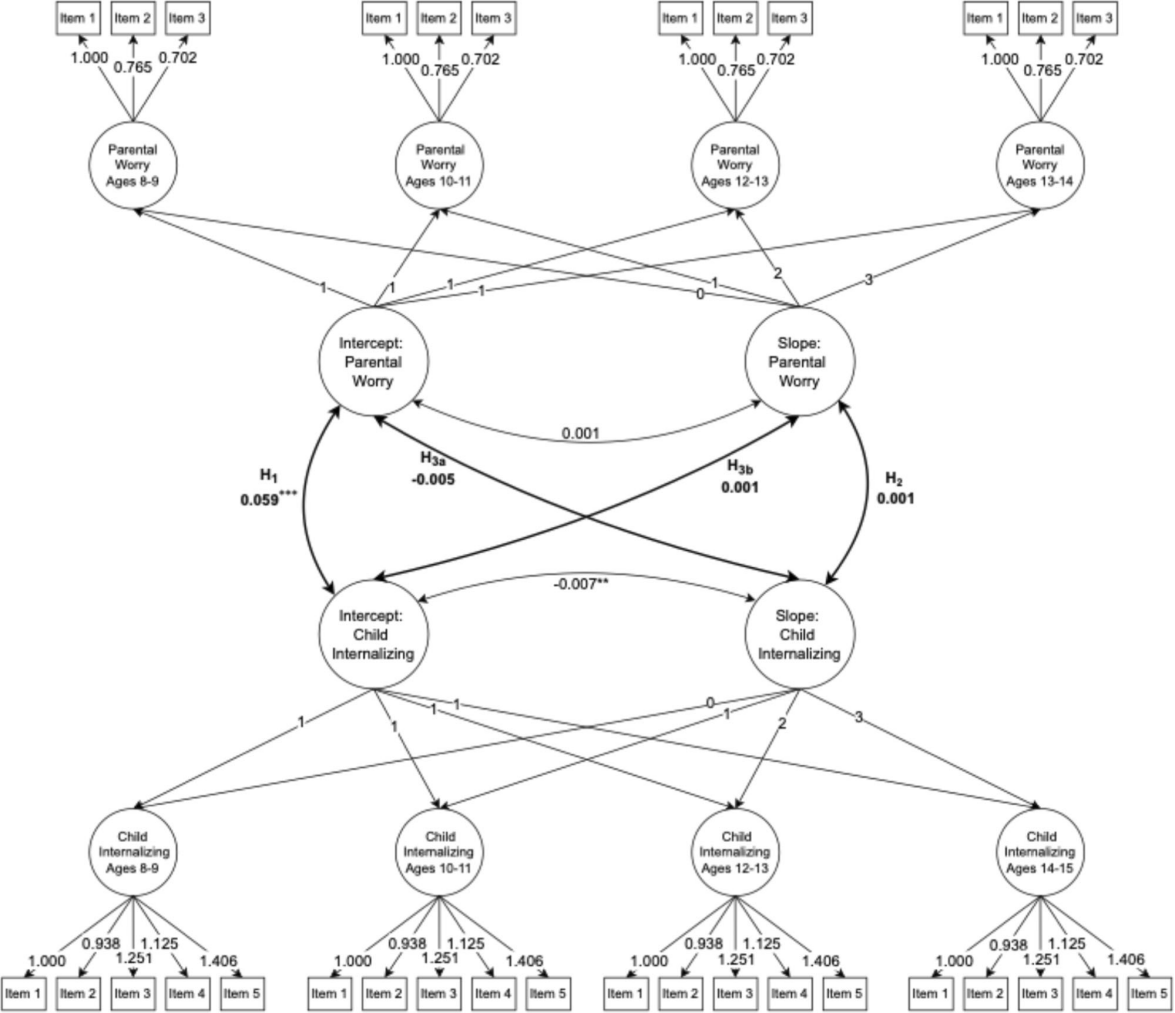
Results of the parallel latent growth model (N = 1260). Hypotheses in bold. The model includes clustered standard errors for the 936 parents. All factor loadings are statistically significant at *p* = 0.001. Otherwise ^*^p < 0.05, ^**^p < 0.01, ^***^p < 0.001

Next, the parental worry and child latent growth models were combined into one model, adding covariances between the parent and child latent intercepts and slopes (see Table 4 and Fig. 2). Model fit was good (RMSEA = 0.021, CFI = 0.976, SRMR = 0.036). In line with Hypothesis 1, parental worry and child internalizing symptoms were positively related when children were ages 8 and 9 (*B* = 0.059, *p* < 0.001). Hence, when the child was in this age group, an increase of one scale point in parental worry was associated with an average increase of 0.059 in child internalizing symptoms. Against Hypothesis 2, there was not a statistically significant positive correlation between the slopes of parental worry and child internalizing symptoms (*B* = 0.001, *p* = 0.763). Similarly, the intercept of parental worry was not related to the slope of child internalizing symptoms (*B* = −0.005, *p* = 0.369), nor was the intercept of child internalizing symptoms related to the slope of parental worry (*B* = 0.001, *p* = 0.777). Thus, the results do not support Hypotheses 3_a-b_.

**Table 4:** Parameter estimates for parallel latent growth model (N = 1260)

Parameter	Unstandardized estimate	Standardized estimate
*Growth factors*		
S: Parental worry	−0.062^***^ (0.014)	−0.809 (0.512)
S: Child internalizing symptoms	−0.017^**^ (0.006)	−0.208^**^ (0.078)
*Growth factor covariances*		
I: Parental worry ↔ I: Child internalizing symptoms	0.059^***^ (0.012)	0.319^***^ (0.060)
S: Parental worry ↔ S: Child internalizing symptoms	0.001 (0.002)	0.080 (0.269)
I: Parental worry ↔ S: Child internalizing symptoms	−0.005 (0.005)	−0.068 (0.076)
I: Child internalizing symptoms ↔ S: Parental worry	0.001 (0.004)	0.063 (0.226)
I: Parental worry ↔ S: Parental worry	0.001 (0.015)	0.008 (0.232)
I: Child internalizing symptoms ↔ S: Child internalizing symptoms	−0.007^**^ (0.003)	−0.399^***^ (0.085)
*Variances*		
I: Parental worry	0.711^***^ (0.059)	1.000 (0.000)
S: Parental worry	0.006 (0.007)	1.000 (0.000)
I: Child internalizing symptoms	0.048^***^ (0.007)	1.000 (0.000)
S: Child internalizing symptoms	0.007^***^ (0.002)	1.000 (0.000)

“I” represents “intercept” and “S” represents “slope”. Standard error in parentheses. The model includes clustered standard errors for the 936 parents and has 438 degrees of freedom. Model fit indices are *x*^2^: 674.163; RMSEA: 0.021; CFI: 0.976; TLI: 0.973; SRMR: 0.036

^*^p < 0.05, ^**^p < 0.01, ^***^p < 0.001

Adding the control variables did not substantially change the model conclusions (see Table S4 and Figure S3). The relation between initial parental worry and child internalizing symptoms reduced slightly (*B* = 0.034, *p* < 0.001), and all other coefficients related to the hypotheses remained close to zero and statistically insignificant. When taking into account potential confounders, the mean of the latent slope for parental worry remained negative, but the effect size decreased slightly and was no longer statistically significant (μ = −0.049, *p* = 0.277). The correlations between control variables and the latent intercept and slope factors indicate whether, and to what extent, baseline levels and change over time are associated with these covariates—for instance, whether developmental trajectories differ by parental gender. The results showed that mothers were, on average, more worried about their children than fathers when their child was 8–9 years old (*B* = 0.378, *p* < 0.001). While girls and boys did not differ substantially in their internalizing symptoms at the ages 8–9 (*B* = −0.026, *p* = 0.169), the average decrease in internalizing symptoms for girls across adolescence was weaker than that of boys (*B* = −0.078, *p* < 0.001). Moreover, higher parental education was associated with lower initial parental worry about one’s children (*B* = −0.181, *p* < 0.001) and lower child internalizing symptoms (*B* = −0.027, *p* < 0.001). However, socioeconomic differences in child internalizing symptoms reduced over time (*B* = 0.010, *p* < 0.001).

### Covariances Over Time

**Fig. 3:**
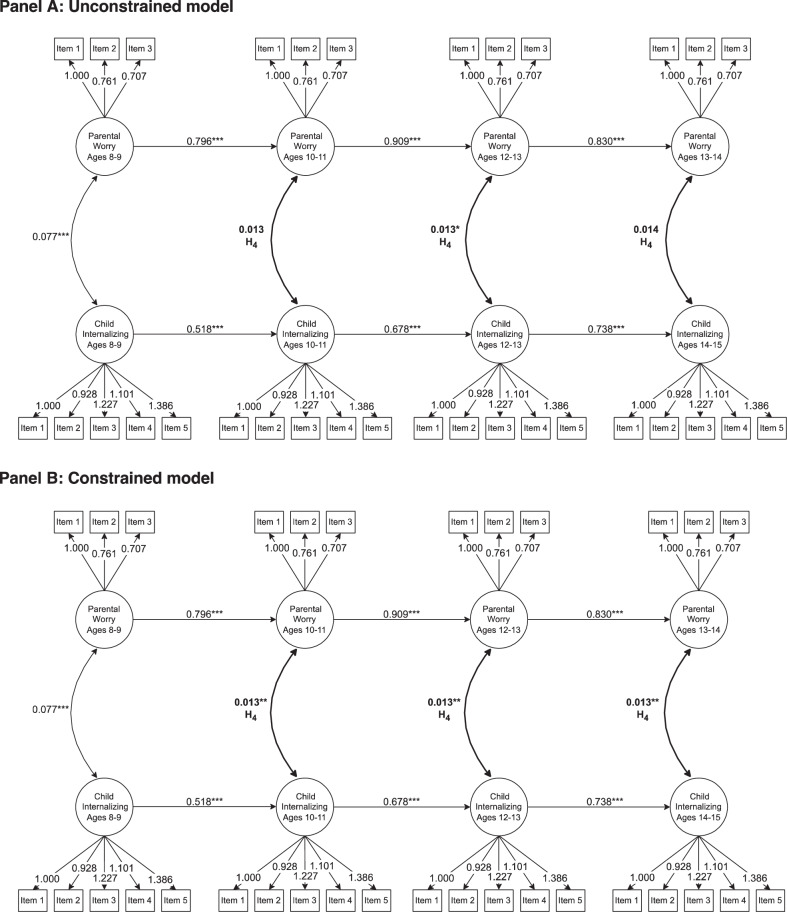
Results of the covariances between parental worry and child internalizing symptoms over time (N = 1260). **A** depicts the unconstrained model, which allows the covariances between parental worry and child internalizing symptoms in age groups 10–11, 12–13, 14–15 to vary over time. **B** shows the constrained model constrains the covariances to be equal. Hypotheses in bold. All factor loadings are statistically significant at *p* = 0.001. Otherwise ^*^p < 0.05, ^**^p < 0.01, ^***^p < 0.001

The next analyses tested whether the covariance between parental worry and child internalizing symptoms changes over time as the child ages (Model 2). Results from the model are presented in Fig. 3 and Table 5. First, an unconstrained model was estimated, allowing the relation between parental worry and child internalizing symptoms to vary over time. When the child was 8–9, the covariance was *B* = 0.077 (*p* < 0.001). Meanwhile, the covariances between parental worry and child internalizing symptom controlling for the autoregressive path, namely the effect of parental worry and child internalizing symptoms of the previous wave, were 10–11 (*B* = 0.013, *p* = 0.079); 12–13 (*B* = 0.013, *p* < 0.048); and 14–15 (*B* = 0.014, *p* = 0.065). Second, the covariances were constrained to be equal over time in the age groups 10–11, 12–13, and 14–15. In this constrained model, the relation was *B* = 0.013 (*p* = 0.002). Constraining the covariances in the latter three age groups only changed the *x*^*2*^ statistic minimally (*x*^*2*^ difference = 0.041), and this difference was statistically insignificant (*p* = 0.980). The results suggest that the relation between parental worry and child internalizing symptoms was largely stable as the child ages, leading to the rejection of Hypothesis 4.

**Table 5:** Changes in covariances between parental worry and child internalizing symptoms over time (N = 1260)

Parameter	Unstandardized estimate	Standardized estimate
**Unconstrained model**		
Ages 8–9: Parental worry ↔ child internalizing symptoms	0.077^***^ (0.013)	0.279^***^ (0.044)
Ages 10–11: Parental worry ↔ child internalizing symptoms	0.013 (0.007)	0.125 (0.068)
Ages 12–13: Parental worry ↔ child internalizing symptoms	0.013^*^ (0.006)	0.116^*^ (0.057)
Ages 14–15: Parental worry ↔ child internalizing symptoms	0.014 (0.007)	0.112 (0.060)
**Constrained model**		
Ages 8–9: Parental worry ↔ child internalizing symptoms	0.077^***^ (0.013)	0.279^***^ (0.043)
Ages 10–11: Parental worry ↔ child internalizing symptoms	0.013^**^ (0.004)	0.124^**^ (0.039)
Ages 12–13: Parental worry ↔ child internalizing symptoms	0.013^**^ (0.004)	0.120^**^ (0.038)
Ages 14–15: Parental worry ↔ child internalizing symptoms	0.013^**^ (0.004)	0.107^**^ (0.035)

The unconstrained model allows the covariances between parental worry and child internalizing symptoms in age groups 10–11, 12–13, 14–15 to vary over time, while the constrained model constrains the covariances to be equal. Standard error in parentheses. Both models include clustered standard errors for the 936 parents. The unconstrained model has 440 degrees of freedom and the model fit indices *x*^2^: 799.020; RMSEA: 0.025; CFI: 0. 964; TLI: 0.960; SRMR: 0.042. The constrained model has 442 degrees of freedom and the model fit indices *x*^2^: 798.914; RMSEA: 0.025; CFI: 0.964; TLI: 0.960; SRMR: 0.042. The *x*^2^ difference test has an *x*^*2*^ difference of 0.041, a degree of freedom difference of 2, and a p-value of 0.980

^*^p < 0.05, ^**^p < 0.01, ^***^p < 0.001

## Discussion

As previously theorized, parental worry about one’s children may be a key link explaining the relation between parental anxiety and child mental health issues (Fisak et al., 2012). Nevertheless, there has been a paucity of empirical evidence examining the role of parental worry. Studies that exist are cross-sectional and do not take a longitudinal, developmental perspective. Therefore, this study sought to fill this gap in the literature, examining the relations among the initial levels (intercept) and trajectories (slope) of parental worry and child internalizing symptoms and testing the stability of the relations over time. The study demonstrated that although parental worry and child internalizing symptoms are related at ages 8–9, the trajectories are not related, and the association remains stable over time.

In doing so, this study extends methodologically past the existing literature. Firstly, the analyses are conducted using parallel latent growth models, approaching the issue of bidirectionality through the lens of developmental trajectories. This approach offers a methodological alternative, considering that the typically used cross-lagged panel models have been shown to produce biased estimates (Hamaker et al., 2015; Lucas, 2023). Secondly, this study operationalizes parental worry and child internalizing symptoms as latent variables, which allows for checking longitudinal method invariance and incorporating measurement error in models. Thirdly, parents and children each provide self-assessments, which safeguard against the possibility of shared method variance (cf. Youngstrom et al., 1999). Moreover, the results are additionally practically relevant due to the widespread rates of anxiety and depression among youth (Castelpietra et al., 2022).

Given the possibility that children affect parents (Pardini, 2008), a great deal of research has sought to disentangle the directionality of parental-child effects (e.g., Allmann et al., 2022). While most research has focused on lagged effects, controlling for previous states and ideally, stable between-person differences, this study took a different perspective, examining bidirectionality in the form of linked initial states and trajectories. The results show that parental worry about one’s children tends to decrease slightly over time, and there is little variation in trajectories. Similarly, child internalizing symptoms decrease on average from late childhood to early adolescence, but there is substantial variation in these trajectories. Hence, the evidence suggests a lack of reactivity to changes in one dyad on the other that can be explained by the lack of variation in the trajectories of parental worry about one’s children. In sum, the results do not suggest “linked lives” or child-to-parent effects on the other’s future respective trajectories.

These results are surprising given related studies examining parental and child mental health trajectories. For example, previous research suggests that maternal mental health shapes children’s mental health trajectories (Wang et al., 2018), and the trajectories of parental and child stress are related (Griffith et al., 2023). However, the former examined a different period of development, focusing on early through late childhood (Wang et al., 2018), while this study examined late childhood through adolescence. Meanwhile, the latter operationalized stress as stressful life events (Griffith et al., 2023); hence, the co-occurring trajectories found in their study may be due to shared life circumstances, rather than mutual mental health influences. Hence, more research is needed to understand the lack of variation in the trajectories of parental worry and the extent to which parents’ and children’s mental health trajectories are linked.

In contrast to competing hypotheses predicting increasing or decreasing associations between parental worry and child internalizing symptoms, the present findings showed a stable relationship across late childhood through adolescence. One possibility is that opposing mechanisms—such as escalating accommodation versus decreasing parental influence—may counterbalance each other, resulting in no overall change. Hence, future research should explicitly test the underlying mechanisms to understand the drivers of the stable association.

While this study focused on the longitudinal relations between parental worry and child internalizing symptoms, the covariates offer additional insights into how initial levels and trajectories of internalizing symptoms varied across sociodemographic groups. The results show boys and girls exhibited similar levels of internalizing symptoms at ages 8–9; however, boys showed a steeper decline in symptoms across late childhood and early adolescence. Thus, this study also adds to the evidence on the widening of a gender gap in internalizing symptoms during adolescence (Katsantonis, 2024; Leve et al., 2005). In addition, the finding that mothers reported higher levels of parental worry than fathers is consistent with prior research showing that women tend to be more anxious than men (McLean & Anderson, 2009) and more often engage in overprotective parenting (Venard et al., 2024). Hence, this study extends on existing findings by demonstrating that such gender differences are also evident in child-specific parental worry.

### Limitations

Despite the several contributions to the literature, this study is subject to limitations. Firstly, this study focused on trajectories; however, other forms of bidirectionality are likely, particularly at the interaction level. It could be that situational-level interactions reinforce the relation in a bidirectional manner, but these patterns reinforce themselves rather than shape each other’s trajectories. For example, the child could regularly display anxious avoidance behavior, which the parent reinforces (Dadds et al., 1996). Such a behavioral pattern would be similar to that described by Patterson (1995) without the escalating tendencies and potentially explain the evidence of stable relations throughout development. Avenues forward include the continued use of observational studies, which have long been used to describe and explain the behaviors of dyads at the situational level (e.g., Dadds & Roth, 2001; Patterson, 1995); Experience Sampling Method, which has shorter time frames of days and weeks between assessments (Jian et al., 2025; Van Berkel et al., 2018); or vignette survey experiments (Aguinis & Bradley, 2014) for disentangling this bidirectionality.

Moreover, the parental worry measure assessed worry about all of one’s children, rather than child-specific parental worry. However, it could be that such parental worry is child-specific (cf. Putnam et al., 2002), whereby parents increase their worry about one child due to their increasing internalizing symptoms. Therefore, future research should continue to examine the extent to which parental worry about one’s children is child-specific and replicate this study with a child-specific measure.

While the wave 1 pairfam sample was designed as a representative cohort panel study, this analytic sample is not representative of the German population. Participation in the child survey required parental consent, and not all children with consent ultimately participated. The sample also includes cases from the East German oversample, which may introduce regional imbalances in representation (Brüderl, Drobnič, et al., 2023). Furthermore, attrition analyses showed that parent-child dyads with mothers and a higher level of parental education were more likely to be included in the final sample. Although this mirrors the tendency for studies investigating child mental health outcomes to focus on maternal behavior (cf. L. B. Jones et al., 2021), future research should jointly consider parental worry of mothers and fathers when explaining child mental health outcomes to examine whether the effects differ by parent gender as well as examine whether the findings hold in more representative samples.

Finally, the dataset does not include genetic information on the parent and child, therefore, it is unable to assess whether the shared covariance of parental worry and child internalizing symptoms over time was due to genetic similarities. Given the known role of genetics in the intergenerational transmission of anxiety (Lebowitz et al., 2016), this limitation highlights the importance of including genetic indicators in social science panel studies.

## Conclusion

Parental worry about one’s children has been overlooked in explaining how parental mental health and parenting behaviors shape child mental health outcomes. This study shines a light on this gap, examining the longitudinal relations between parental worry about one’s children and child internalizing symptoms from late childhood through adolescence. The results demonstrate that while parental worry and child internalizing symptoms are linked at younger ages, their trajectories are not related, nor do initial levels influence the trajectories of the other. Moreover, the relation remains relatively stable across this developmental span. As the results show that parental worry about one’s child and child internalizing symptoms are positively related by ages 8–9 and remain stable throughout adolescence, highlighting the importance of interrupting the intergenerational transmission of anxiety at early ages.

## Supplementary information

Supplementary Information
