# Supplementary material for: Different Trajectories, Stable Links: Parental Worry and Child Internalizing Symptoms Over Time
Source: J Youth Adolesc. 2025 Aug 22;54(10):2524–39. doi: 10.1007/s10964-025-02237-1 (PMC12479578; doi:10.1007/s10964-025-02237-1)
Supplement: Supplementary file 1 — Supplementary Information [file 10964_2025_2237_MOESM1_ESM.docx]

**Table S1: Attrition analyses predicting inclusion in final sample (N = 3,070).**

| **Parameter** | **Odds ratios** |
| --- | --- |
| *Parents* |  |
| Parental worry | 0.909  (0.822; 1.004) |
| Parent female | 1.443^***^  (1.187; 1.755) |
| Parent age | 1.011  (0.990; 1.034) |
| Highest level of education | 1.103^**^  (1.039; 1.171) |
| Migration status (ref. born in Germany) | 0.807  (0.611; 1.068) |
| Monthly net household income | 1.131  (0.929; 1.375) |
| Total number of children | 0.933  (0.852; 1.022) |
| Relationship status (ref. no cohabitating partner) | 1.181  (0.788; 1.770) |
| Currently living in East Germany (ref. West Germany) | 1.125  (0.892; 1.418) |
| East German oversample (ref. in main sample) | 0.908  (0.683; 1.206) |
| *Children* |  |
| Child internalizing symptoms | 0.981  (0.792; 1.214) |
| Child female | 0.874  (0.746; 1.024) |
| Child age wave 1 (pairfam) | 1.316^***^  (1.276; 1.358) |
| Child age first participation | 0.380^***^  (0.349; 0.415) |
| Constant | 199.488^***^  (35.777; 1112.308) |

*Notes:* The first recorded value was used for time-changing variables. Parental worry and child internalizing symptoms averaged to create indices. Confidence intervals in parentheses. The model includes cluster-robust standard errors for the 2,055 parents.

^*^*p*<0.05, ^**^*p*<0.01, ^***^*p*<0.001.

**Table S2: Parameter estimates for parental worry latent growth model (N = 936).**

| **Parameter** | **Unstandardized estimate** | **Standardized estimate** |
| --- | --- | --- |
| *Growth factors* |  |  |
| S: Parental worry | -0.063^***^  (0.016) | -0.698  (0.423) |
| *Growth factor covariances* |  |  |
| I: Parental worry ↔ S: Parental worry | -0.004  (0.017) | -0.053  (0.203) |
| *Variances* |  |  |
| I: Parental worry | 0.695^***^  (0.059) | 1.000  (0.000) |
| S: Parental worry | 0.008  (0.009) | 1.000  (0.000) |

*Notes*: “I” represents “intercept” and “S” represents “slope”. Standard error in parentheses. The model has 44 degrees of freedom. Model fit indices are *x*^2^: 71.591; RMSEA: 0.026; CFI: 0.993; TLI: 0.989; SRMR: 0.029.

^*^*p*<0.05, ^**^*p*<0.01, ^***^*p*<0.001.

**Table S3: Parameter estimates for child internalizing symptoms latent growth model (N = 1,260).**

| **Parameter** | **Unstandardized estimate** | **Standardized estimate** |
| --- | --- | --- |
| *Growth factors* |  |  |
| S: Child internalizing symptoms | -0.017^**^  (0.006) | -0.208^**^  (0.078) |
| *Growth factor covariances* |  |  |
| I: Child internalizing symptoms ↔ S: Child internalizing symptoms | -0.007^**^  (0.003) | -0.399^***^  (0.086) |
| *Variances* |  |  |
| I: Child internalizing symptoms | 0.048^***^  (0.007) | 1.000  (0.000) |
| S: Child internalizing symptoms | 0.007^***^  (0.002) | 1.000  (0.000) |

*Notes*: “I” represents “intercept” and “S” represents “slope”. Standard error in parentheses. The model includes clustered standard errors for the 936 parents and has 158 degrees of freedom. Model fit indices are *x*^2^: 341.003; RMSEA: 0.030; CFI: 0.948; TLI: 0.938; SRMR: 0.041.

^*^*p*<0.05, ^**^*p*<0.01, ^***^*p*<0.001.

**Table S4: Parameter estimates for parallel latent growth model with control variables (N = 1,260).**

| **Parameter** | **Unstandardized estimate** | **Standardized estimate** |
| --- | --- | --- |
| *Growth factors* |  |  |
| S: Parental worry | -0.049  (0.045) | -0.607  (0.625) |
| S: Child internalizing symptoms | -0.097^***^  (0.020) | -1.162^***^  (0.254) |
| *Growth factor covariances* |  |  |
| I: Parental worry ↔ I: Child internalizing symptoms | 0.043^***^  (0.010) | 0.280^***^  (0.066) |
| S: Parental worry ↔ S: Child internalizing symptoms | 0.000  (0.002) | 0.086  (0.282) |
| I: Parental worry ↔ S: Child internalizing symptoms | 0.000  (0.005) | 0.006  (0.089) |
| I: Child internalizing symptoms ↔ S: Parental worry | 0.001  (0.004) | 0.076  (0.219) |
| I: Parental worry ↔ S: Parental worry | 0.003  (0.014) | 0.045  (0.254) |
| I: Child internalizing symptoms ↔ S: Child internalizing symptoms | -0.005^*^  (0.002) | -0.367^*^  (0.102) |
| *Residual variances* |  |  |
| I: Parental worry | 0.554^***^  (0.052) | 0.794^***^  (0.033) |
| S: Parental worry | 0.006  (0.007) | 0.978^***^  (0.048) |
| I: Child internalizing symptoms | 0.043^***^  (0.007) | 0.945^***^  (0.022) |
| S: Child internalizing symptoms | 0.005^**^  (0.002) | 0.726^***^  (0.067) |
| *Control variables* |  |  |
| Child female → I: Parental worry | -0.002  (0.056) | -0.001  (0.033) |
| Parent female → I: Parental worry | 0.378^***^  (0.063) | 0.209^***^  (0.035) |
| Child age wave 1 → I: Parental worry | 0.029^*^  (0.012) | 0.088  (0.036) |

| Parental education→ I: Parental worry | -0.181^***^  (0.020) | -0.364^***^  (0.039) |
| --- | --- | --- |
| Child female → I: Child internalizing symptoms | -0.026  (0.019) | -0.061  (0.045) |
| Parent female → I: Child internalizing symptoms | -0.023  (0.021) | -0.049  (0.045) |
| Child age wave 1 → I: Child internalizing symptoms | 0.005  (0.004) | 0.061  (0.049) |
| Parental education→ I: Child internalizing symptoms | -0.027^***^  (0.006) | -0.214^***^  (0.046) |
|  |  |  |
| Child female → S: Parental worry | -0.002  (0.018) | -0.014  (0.115) |
| Parent female → S: Parental worry | -0.008  (0.020) | -0.047  (0.118) |
| Child age wave 1 → S: Parental worry | -0.004  (0.005) | -0.129  (0.160) |
| Parental education→ S: Parental worry | 0.002  (0.006) | 0.035  (0.035) |
|  |  |  |
| Child female → S: Child internalizing symptoms | 0.078^***^  (0.010) | 0.463^***^  (0.062) |
| Parent female → S: Child internalizing symptoms | 0.012  (0.009) | 0.067  (0.050) |
| Child age wave 1 → S: Child internalizing symptoms | -0.004  (0.002) | -0.111  (0.064) |
| Parental education→ S: Child internalizing symptoms | 0.010^***^  (0.003) | 0.207^***^  (0.054) |

*Notes*: “I” represents “intercept” and “S” represents “slope”. The model includes clustered standard errors for the 936 parents and has 550 degrees of freedom. Model fit indices are *x*^2^: 848,971; RMSEA: 0.021; CFI: 0.972; TLI: 0.968; SRMR: 0.036.

^*^*p*<0.05, ^**^*p*<0.01, ^***^*p*<0.001.

**Fig. S1**

*Notes*: Visualization of parental worry latent growth model results (N = 936). All factor loadings are statistically significant at *p* = 0.001.

Otherwise ^*^*p*<0.05, ^**^*p*<0.01, ^***^*p*<0.001.

**Fig. S2**

*Notes*: Visualization of child internalizing symptoms latent growth model results (N = 1,260). The model includes clustered standard errors for the 936 parents. All factor loadings are statistically significant at *p* = 0.001.

Otherwise ^*^*p*<0.05, ^**^*p*<0.01, ^***^*p*<0.001.

**Fig. S3**

*Notes*: Visualization of parallel latent growth model results (N = 1,260). Hypotheses in bold. The model includes clustered standard errors for the 936 parents. Only statistically significant pathways for covariates shown. All factor loadings are statistically significant at *p* = 0.001.

Otherwise ^*^*p*<0.05, ^**^*p*<0.01, ^***^*p*<0.001.
